# Clinical Features, Histopathology and Differential Diagnosis of Sarcoidosis

**DOI:** 10.3390/cells11010059

**Published:** 2021-12-26

**Authors:** Claudio Tana, Iginio Donatiello, Alessandro Caputo, Marco Tana, Teresa Naccarelli, Cesare Mantini, Fabrizio Ricci, Andrea Ticinesi, Tiziana Meschi, Francesco Cipollone, Maria Adele Giamberardino

**Affiliations:** 1 Geriatrics Clinic, SS. Medical Department, SS. Annunziata Hospital of Chieti, 66100 Chieti, Italy; 2 Internal Medicine Unit, Medical Department, University Hospital of Salerno, 84121 Salerno, Italy; iginiodonatiello@gmail.com; 3 Anatomical Pathology Unit, Department of Anatomical Pathology, University Hospital of Salerno, 84121 Salerno, Italy; alcap94@gmail.com; 4 2nd Internal Medicine Unit, SS. Medical Department, SS. Annunziata Hospital of Chieti, 66100 Chieti, Italy; marco_tana@yahoo.it; 5 Oncoematology Unit, Oncoematology Department, Tor Vergata Hospital of Rome, 00133 Rome, Italy; teresanaccarelli@gmail.com; 6 Department of Neuroscience, Imaging and Clinical Sciences, Institute of Radiology, SS. Annunziata Hospital of Chieti, 66100 Chieti, Italy; cesare.mantini@gmail.com (C.M.); fabrizioricci@hotmail.it (F.R.); 7 Internal Medicine Unit, Geriatric-Rehabilitation Department and Department of Medicine and Surgery, University of Parma, Via Antonio Gramsci 14, 43126 Parma, Italy; andrea.ticinesi@unipr.it (A.T.); tiziana.meschi@unipr.it (T.M.); 8 Department of Medicine and Science of Aging, Medical Clinic, SS Annunziata Hospital of Chieti, G. D’Annunzio University of Chieti, 66100 Chieti, Italy; fcipollone@unich.it; 9 Department of Medicine and Science of Aging and CAST, Geriatrics Clinic, SS. Annunziata Hospital of Chieti, G. D’Annunzio University of Chieti, 66100 Chieti, Italy; mag@unich.it

**Keywords:** sarcoidosis, granuloma, histopathology, cell, diagnosis, medicine

## Abstract

Sarcoidosis is a chameleon disease of unknown etiology, characterized by the growth of non-necrotizing and non-caseating granulomas and manifesting with clinical pictures that vary on the basis of the organs that are mainly affected. Lungs and intrathoracic lymph nodes are the sites that are most often involved, but virtually no organ is spared from this disease. Histopathology is distinctive but not pathognomonic, since the findings can be found also in other granulomatous disorders. The knowledge of these findings is important because it could be helpful to differentiate sarcoidosis from the other granulomatous-related diseases. This review aims at illustrating the main clinical and histopathological findings that could help clinicians in their routine clinical practice.

## 1. Introduction

Sarcoidosis has a heterogeneous presentation, since it can involve every organ and tissue, though lungs and intrathoracic lymph nodes are the sites that are most commonly involved. Clinical onset can be asymptomatic or manifest with acute, subacute or chronic form [1].

Recent data about epidemiology show that Sweden is the country at the highest incidence and prevalence (respectively, 11 and 160 per 100,000 inhabitants), while it is rarely found in countries such as Japan and South Korea (1–5 per 100,000) [2] The worst prognosis is found in African Americans, since they can have an early cardiac involvement with high risk of complications, hospitalization and mortality [3]. Furthermore, black people can more frequently experience a multiorgan involvement and an aggressive form of pulmonary disease [4].

Some forms, such as Lofgren syndrome, a clinical picture characterized by hilar lymph nodes enlargement, erythema nodosum, symmetrical arthritis of the ankles and often anterior uveitis, are specific and virtually diagnostic of an acute form of sarcoidosis [5]. Heerfordt–Waldenstrom syndrome is instead characterized by the presence of Bell’s palsy, fever, anterior uveitis and enlargement of the parotid glands. Both conditions are acute forms of sarcoidosis, as symptoms develop suddenly and usually resolve within a short period (usually a few months) [6].

Subacute onset of sarcoidosis is characterized instead by vanished and non-specific signs and symptoms such as fever, asthenia, weight loss and peripheral lymphadenopathies for less than two years. Here differential diagnosis is impossible on the basis of the clinical picture only [7].

Almost two-thirds of patients with sarcoidosis have an acute course of disease and may undergo spontaneous resolution [8], while 10–30% of cases can become chronic (a duration of disease longer than two years) with a progressive involvement of the lungs and a reduction of pulmonary function. Risk factors are the age of onset for the disease >40 years, black race, extrapulmonary involvement such as heart, skeletal system and eyes with chronic uveitis [9].

## 2. Clinical Pictures of Sarcoidosis

The traditional diagnostic criteria for sarcoidosis have been based on the triad of suggestive clinical picture, non-necrotizing granulomas found at biopsy samples and exclusion of other causes of granulomatous tissue [10]. Although this diagnostic approach is particularly useful for the detection of pulmonary sarcoidosis, it does not resolve the issue of detecting the extrapulmonary involvement, in particular if it is isolated (without lung involvement). For these reasons other diagnostic methods have been used, in particular the WASOG Sarcoidosis Organ Assessment Instrument, that calculates the likelihood of sarcoidosis organ involvement according to a Delphy study methodology and expert agreement consensus [11]. The recent introduction of some advanced imaging techniques, such as deep learning software and algorithms based on artificial-intelligence recognition of imaging findings, showing a similar capacity to humans in recognizing some diagnostic patterns, has provided promising results in the diagnostic approach of patients with sarcoidosis, in particular for those with extra-pulmonary involvement [12,13].

### 2.1. Lung Involvement

In sarcoidosis lungs are affected in 90% of cases, and involvement of mediastinal (hilar) lymph nodes is almost always present [14]. Respiratory symptoms are nonspecific, the most common are dyspnea and cough, but wheezing and chest pain may also be present [15]. Chest X-ray (CXR) and computed tomography (CT) give an important support to the diagnosis of sarcoidosis affecting lungs. The Scadding classification of chest X-rays, including four stages of disease according to the severity of parenchymal and lymph nodes involvement, has been used for staging the lung damage in patients with sarcoidosis (Table 1). However, CXR can underestimate the disease’s extent, while CT has a higher diagnostic accuracy, because it can also detect the smallest parenchymal involvement, that is not otherwise evident on basic radiographs [16]. Typical CT findings are bilateral and hilar lymph node enlargement, nodules with perilymphatic distribution along interlobular septa, subpleural areas and bronchial vessels can confluence into large opacities [17].

Sarcoid granulomas are most frequently located along the perilymphatic regions and along the bronchoscular branches [18]. The airways involvement may be visible macroscopically at bronchoscopy, in fact the bronchial mucosa has alterations such as erythema and a cobblestone appearance and the biopsy in these sites confirms the presence of sarcoid granulomas [19].

Even the pleura, especially the visceral side, can be involved in sarcoidosis by manifesting in most cases with pleural effusion (exudate) and more rarely with pleural masses [20].

In advanced stages, fibrotic and cystic changes can involve the lungs extensively and these alterations can translate into a restrictive ventilatory deficiency at spirometry [21].

Distortion and compression of vessels due to fibrotic changes and sarcoid granulomas can be associated with pulmonary hypertension [22]. Aspergilloma is another complication of sarcoidosis than can manifest with hemoptysis [23].

### 2.2. Extrapulmonary Disease

#### 2.2.1. Eye Disease

Uveitis is the most common clinical presentation and can be anterior or posterior, its clinical detection is important to avoid permanent damage to sight [24].

Anterior uveitis is an inflammation process of the portion that goes from the iris to the ciliary body and clinically manifests with a red, painful eye and photophobia. with a slit lamp examination it is possible to detect keratin precipitates floating in the anterior chamber of the eye [25].

Posterior uveitis is less common and can manifest with decreased vision due to the presence of papilledema and vitreous hemorrhages. The nodules of the choroid give a characteristic appearance called wax drops [26].

Sarcoid conjunctivitis is characterized by the presence of small yellowish and translucent nodules on the conjunctiva, particularly affecting the lower fornix. Sarcoid conjunctivitis occurs only in 5% of affected patients [27].

A particular ophthalmological clinical picture that can accompany sarcoidosis is that of Fuchs’ heterochromic uveitis (FHU); it is a particular form of chronic uveitis, characterized by the development of numerous iris nodules which, subsequently, are accompanied by keratin precipitates and peripheral retinal periplebitis [28].

Lacrimal gland involvement manifests with keratoconjunctivitis sicca, causing itching and burning in the eye with the sensation of a foreign body and swelling of the gland itself [29].

#### 2.2.2. Skin Involvement

Skin manifestations affect 25% of patients with sarcoidosis, and the two most well-known presentations are represented by erythema nodosum and lupus pernio [30,31]. Erythema nodosum is a non-granulomatous and non-caseating panniculitis characterized by the presence of nodules with a soft consistency and overlying red skin that occur on the extensor surface of the legs. However, erythema nodosum is not an exclusive clinical manifestation of sarcoidosis, as it can be found in other diseases such as tuberculosis, Crohn’s disease, rheumatoid arthritis and other autoimmune diseases [32].

Lupus pernio is instead characterized by a rash consisting of bluish-red or purplish lumps that are located on the nose, cheeks, ears, and fingers. Lupus pernio is often associated with severe lung involvement and indicates a poor prognosis [9].

Lupus pernio is characterized by granulomatous vasculitis at the histological examination; it manifests as an organized infiltrate of histiocytes and, above all, of lymphocytes in the reticular dermis, in the perivascular site, with compression of the vascular structures and development of confluent ischemic areas [33].

Other chronic skin lesions consist of plaques or nodules located mainly in the trunk and limbs. Furthermore, the development of granulomas on old scars is not uncommon [34].

#### 2.2.3. Heart

Cardiac sarcoidosis is clinically evident in 2–10% of patients with sarcoidosis, though occult involvement is more frequent as demonstrated by autopsy studies [35].

Although clinically silent, cardiosarcoidosis represents a major cause of death with a mortality rate that can be greater than 85% in autopsy studies. The symptoms and signs of cardiac sarcoidosis depend on the location and extent of the granulomatous inflammation that can affect the myocardium, endocardium, pericardium, and the valvular apparatus [36].

The most frequent manifestations are conduction anomalies including complete atrioventricular block and tachyarrhythmias including ventricular arrhythmias (ventricular or non-sustained tachycardia and ventricular extrasystoles), triggered by anomalous activity of the myocardium or reentry arrhythmias. Other arrhythmias have also been reported such as ectopic atrial activity, paroxysmal atrial tachycardia, atrial flutter and atrial fibrillation [37]. Heart failure (both systolic and diastolic disfunction) is observed in cases of extended granulomatous infiltration of the myocardium, cardiac fibrosis and sudden death [38]. Sudden death from ventricular tachyarrhythmias or conduction blockages has been considered the cause of death in up to 65% of cardiac sarcoidosis cases [39]. Echocardiographic abnormalities can include some nonspecific findings such as wall thickening, segmental abnormalities or ventricular aneurysms. Cardiac magnetic resonance imaging (cMRI) can instead reveal with higher accuracy the presence of edema as gadolinium uptake at T2-weighted images, and findings of chronic disease such as thinning of basal septum wall, ventricular dilatation and systolic dysfunction [3].

The differential diagnosis of sarcoid cardiopathy should always be considered in young or adult persons with cardiac symptoms and those having a diagnosis of sarcoidosis and clinical picture characterized by arrhythmias, conduction abnormalities or heart failure [35].

#### 2.2.4. Liver

The hepatic involvement in sarcoidosis, more frequent in the African American male population, is most often asymptomatic and can manifest only with hepatomegaly and, in 10% of cases, with an increase of transaminases and alkaline phosphatase [40].

Less frequently, hepatic damage from sarcoidosis can evolve toward clear nodules or overt cirrhosis, liver dysfunction and portal hypertension with varices, hepato-pulmonary syndrome and refractory hypoxemia (1% of cases) [41,42].

Hepatic sarcoidosis is characterized histologically by the presence of numerous scattered non-necrotizing granulomas, located more often in the portal spaces. The microscopic structure of the granuloma is the same as that observed in the other organs [43].

#### 2.2.5. Spleen

Spleen involvement in sarcoidosis usually manifests with asymptomatic splenomegaly, while platelet sequestration and thrombocytopenia are less frequently observed. Splenomegaly is rarely so important to lead to splenectomy [44].

Sometimes splenic nodules can be observed at imaging such as ultrasound, and appear as hypoechoic and vascular rounded lesions, that can be misdiagnosed with those observed in malignant processes such as lymphomas [45]. In such cases the differential diagnosis can be very difficult, and biopsy may be the only solution to reveal the presence of granulomatous tissue [46,47,48].

At histological examination, the sarcoid granulomas are small greyish or yellowish-white lesions macroscopically, usually sized less than 1 mm in diameter and similar to miliary tubercles, though unlike miliary tubercles they are more accentuated and have defined margins.

#### 2.2.6. Neurosarcoidosis

Neurosarcoidosis can affect both the central and the peripheral nervous system (CNS and PNS, respectively). The most frequent localizations are leptomeninges, with symptoms similar to other forms of meningitis such as fever, headache and neck rigidity [49]. CSF analysis in these patients shows negative culture, lymphocyte pleocytosis with proteinorrachia and hypoglycorrachia [50].

Macroscopically, nodular lesions have raised appearance, gray-yellowish color and hard consistency, and are sized a few millimeters in diameter. The brain involvement is instead characterized by lesions of various sizes that are located at the periventricular white matter, appearing very similar to the lesions observed in multiple sclerosis. Symptoms depend on the location of these lesions. Brain granulomas are usually larger than those that occur in meningeal granulomatosis; their solid nature and grayish-white appearance often make the differential diagnosis difficult with respect to neoplastic brain lesions, especially gliomas [51].

Other common sites involved in neurosarcoidosis are the cranial nerves, especially the seventh, this can clinically manifest with Bell’s palsy (also bilateral), while the involvement of the optic nerve is associated with visual impairment [52].

Less frequently, the peripheral nervous system may be affected due to both the isolated infiltration of the nerves from epithelioid cells and the development of well-structured granulomatous lesions. Both small and large nerve fibers can be affected causing mononeuropathies, polyradiculopathies and distal and symmetrical polyneuropathies [53].

The infiltration of the pituitary region by sarcoid granulomas can be associated with diabetes insipidus, amenorrhea and galactorrhea [54].

#### 2.2.7. Skeletal System

Bone involvement is reported with a frequency between 1% and 13%, but these percentages are likely to be an underestimation because this localization is usually asymptomatic [55].

Bone lesions mainly affect the phalanges of the hands and feet and the metacarpal and metatarsal bones [56]. The typical histological finding of bone sarcoidosis is Jüngling’s cystoid (Jüngling’s cystoid osteitis multiple). Neville et al. have described three histological types of bone lesions: (a) lytic, rounded or oval lesions located in the central epiphyseal area, particularly at the level of the hand bones; (b) permeative, this type of injury shows a tendency to bone cavitation (“bone tunneling”), with remodeling of the trabecular and cortical architecture and widening of the Havers canals; and (c) destructive, with rapidly developing lesions associated with pathological fractures and frequent joint involvement [57].

The lesions usually involve the cancellous bone tissue and spare the periosteum; in rare cases there is an extension of the process with the involvement of the adjacent joint structures. These lesions are a consequence of the osteodestructive and lytic action of the sarcoid process, rather than a result of bone resorption. Joint involvement manifests with swelling and arthralgia. Overt arthritides are rare and mainly affect large joints, usually ankles in the Lofgren’s syndrome [58]. Arthritides can have a migratory character and are generally transient but sometimes have a chronic course and cause deformities. Sacroiliitis is rare and isolated and can be associated with ankylosing spondylitis which can co-occur with sarcoidosis, especially in HLA B27 positive patients [59].

#### 2.2.8. Bone Marrow Involvement

Bone marrow infiltration from sarcoid granulomas has been exceptionally reported in the literature (incidence of 0.3–2% at biopsy), and can be suggested by variable cytopenia at laboratory exams [60]. More frequently, the presence of cell reduction in particular anemia is most frequently associated with iron deficiency and chronic inflammation, while the presence of leukopenia can derive from hypersplenism, lymphocyte redistribution or can be the effect of immunosuppressive agents [61]. A useful approach to a sarcoidosis patient with cytopenia can be based on a complete blood collection (entire blood cell count, iron and vitamin B12, folate and LDH levels), exclusion of infectious disorders (HCV, HIV, tuberculosis and fungal infections) and, in second line, peripheral smear and bone marrow biopsy when there is a high suspicion of granulomatous infiltration of bone marrow [62].

#### 2.2.9. Renal Disease

Renal involvement in sarcoidosis can manifest with kidney stones and nephrocalcinosis due to increased calcium reabsorption, hypercalciuria and altered calcium metabolism related to the increased production of 1-alpha-hydroxylase by activated macrophages present in granulomas, which causes an increased synthesis of Vitamin D [63].

Renal sarcoid granulomas can be found in both cortical and medullary compartments. Sarcoid nodules can sometimes reach a diameter of 1 cm, thus becoming difficult to differentiate from neoplastic lesions [64].

The histological sites of renal sarcoidosis should be differentiated into interstitial and glomerular. Granulomatous interstitial nephropathy affects approximately 20% of patients with sarcoidosis and represents the most typical histopathological finding of nephrosarcoidosis. Acute and clinically overt renal failure that leads to hemodialysis is uncommon and derives most frequently from an acute granulomatous tubulointerstitial nephritis [63]. Glomerular involvement in sarcoidosis is not common and the mechanism of glomerular damage is not completely known; an autoimmune etiology has been hypothesized because some patients with sarcoidosis and crescent glomerulonephritis or glomerular interstitial nephropathy are positive for antibodies directed against the cytoplasm of neutrophils [64].

## 3. Histopathology of Sarcoidosis

### 3.1. Common Findings

A correct diagnosis of sarcoidosis is suggested by clinical and radiological context, but it is usually reached by showing the presence of noncaseating granulomas at biopsy. These granulomas affect several organs, such as the lungs, skin, spleen, lymph nodes, eye, bone tissue, salivary and lacrimal glands, striated muscles, CNS, endocrine glands and liver, and a correct site of biopsy should be chosen on the basis of the best accessible site and at the lowest risk of complications [65].

Granulomatous inflammation is a well-defined chronic inflammatory process, in which the activated macrophage is the predominant cell and has the appearance of epithelial cells, taking for this reason the name of “epithelioid cell”. Granuloma is a circumscribed and well-organized area of granulomatous inflammation, formed by epithelioid cells, lymphocytes, leukocytes and, sometimes, plasma cells. Epithelioid cells can at times merge to create giant cells, which are sometimes located in the periphery, sometimes in the center of the granuloma. Giant cells can reach a diameter of about 50 microns and are composed of abundant cytoplasm containing over 20 small nuclei that are peripherally localized (Langherans-type giant cells) or dispersed in the cytoplasm (foreign body-like giant cells) [43].

Lymphocytes appear more numerous in stages and surround clusters of epitheliod cells. In the initial stages, CD4+ T helper cells are found, and facilitate the formation and maintenance of the granulomatous lesion through the release of specific cytokines [66].

**Figure 1:**
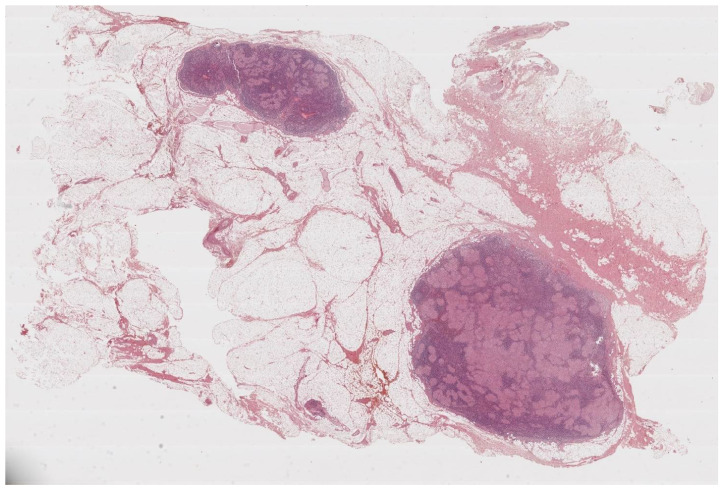
Lymph nodes involved in sarcoidosis. The characteristic low-power appearance of lymph nodal sarcoidosis consists of diffuse effacement of the nodal architecture by small and regular non-caseating granulomas. The pink, eosinophilic granulomas stand in stark contrast against the overall basophilic look of the lymph nodes. (Haematoxylin and eosin, 5×).

**Figure 2:**
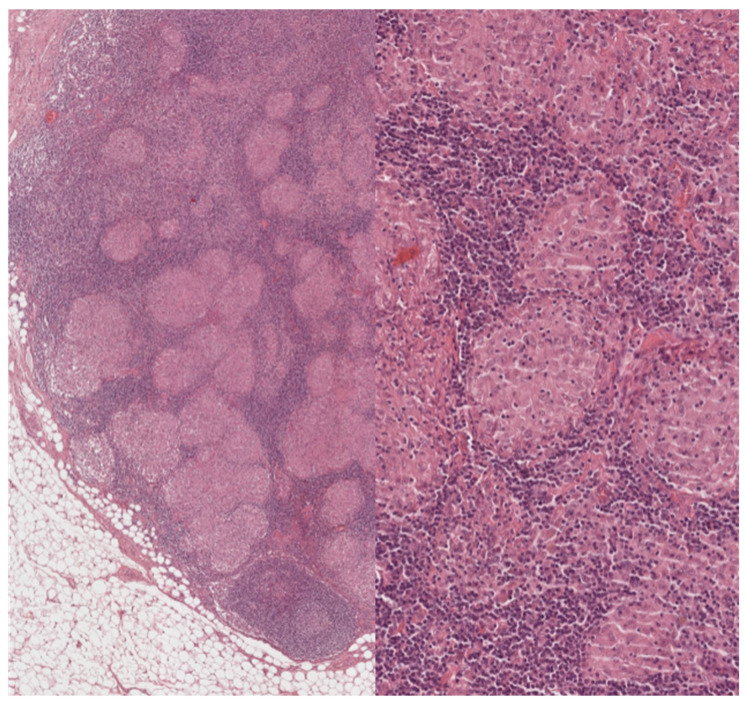
Lymph nodes involved in sarcoidosis: high-power features. On higher power, the granulomas appear eosinophilic and of regular size, and no caseation necrosis is evident. Sarcoid granulomas characteristically recruit few if any lymphocytes. In a lymph node, however, this aspect is not easy to assess because the normal parenchyma is constituted by densely packed lymphocytes. Within the granulomas, however, lymphocytic infiltration is minimal. (Haematoxylin and eosin, 40× and 200×).

Subsequently, the number of T helper lymphocytes of the granuloma reduce, favoring an increase of T CD8+; the increase of cytotoxic lymphocytes is associated with a tendency to the resolution of the granulomatous lesion (Figure 1 and Figure 2) [66].

**Figure 3:**
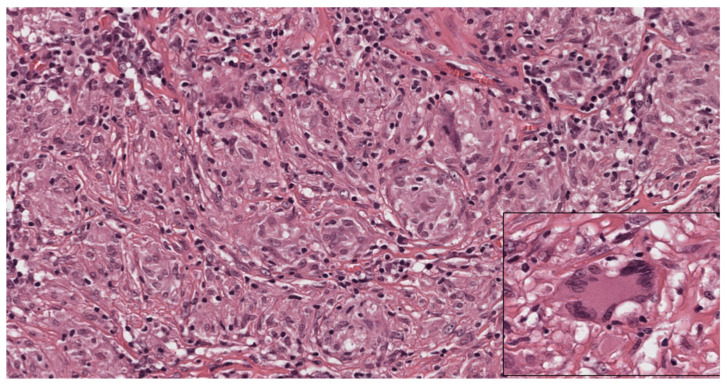
Cytological aspects of sarcoidosis. The granulomas are composed mostly of epithelioid histiocytes with sparse intermingled mature lymphocytes. The histiocytes show oval nuclei and abundant eosinophilic cytoplasm. Occasional giant cells (inset) can be observed. (Haematoxylin and eosin, 300×; inset: 400×).

The number of plasma cells is usually very low; their presence indicates an increase of local humoral immunity (Figure 3).

The natural tendency of the sarcoid granulomatous lesion is towards healing; most inflammatory cells tend to disappear. In other cases, the granuloma evolves towards the development of typical rings of collagen fibers at the periphery of the lesion (ring fibrosis), which are then replaced by a hyaline and dense scar [67,68].

Scar tissue, the result of the previous granuloma, can sometimes persist over time; the tendency to persistent fibrosis is more typical of some organs such as the skin and lungs. Fibrosis can sometimes accompany the deposit of substances such as oxalate or calcium carbonate [69].

**Figure 4:**
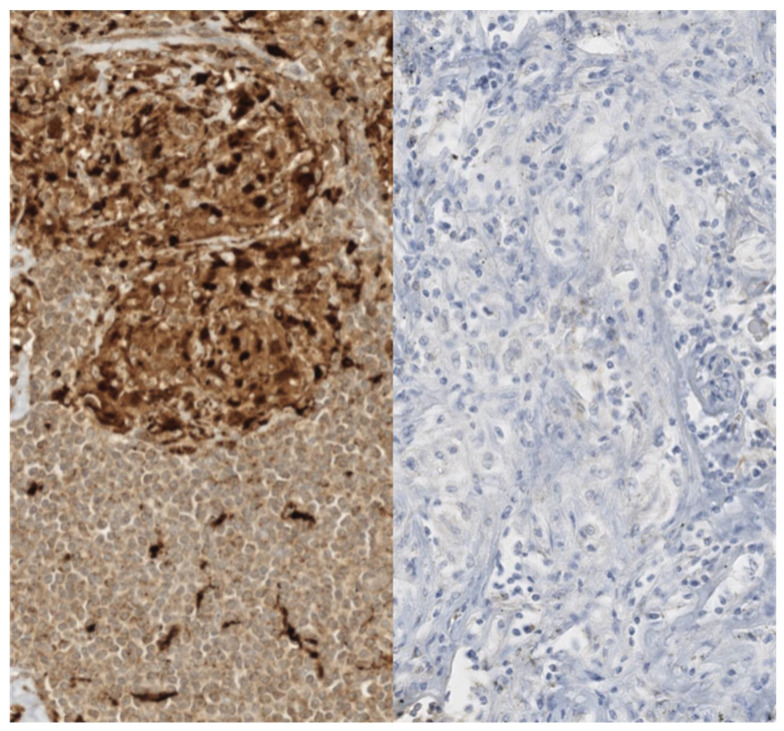
Immunohistochemical aspects of sarcoid granulomas. The histiocytes constituting sarcoid granulomas are strongly reactive for histiocytic markers such as CD68 (left). A pan-cytokeratin stain (AE1/AE3, right) should be performed and should be negative. Dense granulomatous inflammation can sometimes hide a neoplasm completely.

A central area of fibrinoid necrosis can be present in 6–35% of cases, this area of necrosis is distinguishable from caseous necrosis due to the persistence of an intact reticular pattern [70]. The presence of dense granulomatous inflammation can sometimes hide a neoplasm, that could be revealed by immunohistochemical analysis and performing a pan-cytokeratin stain (Figure 4).

Some inclusion bodies can be found inside granulomas and can lead to a suspicion of sarcoidosis, though they are not pathognomonic of it. These bodies are the Schaumann (also called conchoidal) bodies, laminated and birefringent concretions consisting of calcium and proteins usually in the cytoplasm of giant cells [71], asteroid bodies which are stellar-shaped inclusions present in the cytoplasm of giant cells (only in 2–9% of sarcoid granulomatous lesions), refractive but not birefringent under a polarizing microscope [67], and the Hamazaki-Wesenberg bodies which are bodies with variable shape from oval to fusiform of about 0.5 microns in size and brownish in color of likely lysosomal origin. Their structure is not very different from those of ceroid and lipofuscin-like pigments [72].

### 3.2. Other Histopathological Findings

In addition to the classic non-necrotizing form, sarcoidosis can give other histopathological types, called necrotizing sarcoid granulomatosis and nodular sarcoidosis.

Necrotizing sarcoid granulomatosis (NSG) is a rare and controversial histopathological picture, it is still debated whether to consider it a form of sarcoidosis or a distinct pathological entity [73]. It mainly affects the lungs and is characterized by the association of necrotizing sarcoid granulomas with lymphocytic vasculitis [67].

**Figure 5:**
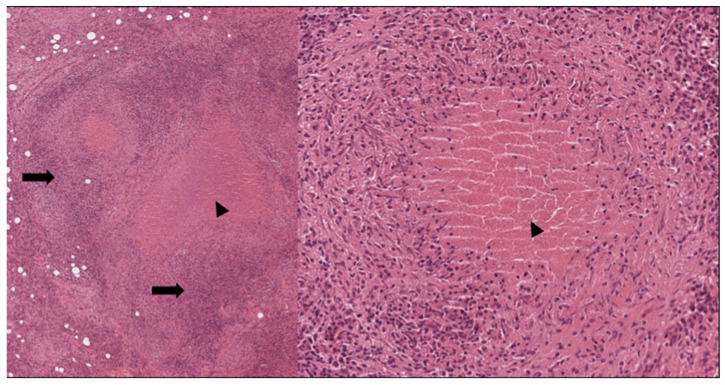
Necrotizing granulomas. Note the central eosinophilic necrosis (arrowhead) and the abundant lymphocytes (arrows); (Left: H&E 40×; right: H&E 200×).

The granulomatous lesions can sometimes involve the lymphatic tracts or be associated with an interalveolar granulomatous component. A characteristic of necrotizing sarcoid granulomatosis is the presence of extensive areas of necrosis and vasculitis, mainly involving the muscular arteries and pulmonary veins, characterized by the presence of necrotizing giant cell granulomas or by the infiltration of lymphocytes and macrophages in the vessel wall (Figure 5) [74].

Such necrotic areas can give diagnostic issues with those found in granulomas associated with infectious agents, especially mycobacteria [75].

Nodular sarcoidosis is instead a histological variant characterized by the presence of non-necrotizing granulomas confluent in large masses, often accompanied by extensive fibrosis of the contiguous tissues. The prevalence of this histological variant of benign lymphogranulomatosis ranges from 2% to 4% of patients with sarcoidosis. Nodular sarcoidosis usually affects the lung parenchyma, and nodular lesions with a diameter of 1–5 cm are described, rarely they can reach a size of 7 cm. Nodular sarcoidosis can sometimes manifest as a solitary, ‘tumor-like’ mass. This variant of sarcoidosis is usually accompanied by hilar lymphadenopathy and affects young patients, aged between 20 and 40 years [67].

## 4. Differential Diagnosis of Sarcoid Granulomas

Although sarcoid granuloma is distinctive, it is not pathognomonic of sarcoidosis being common also in other non-necrotizing granulomatous disorders [76]. Some disorders can share some typical histopathological findings or present with more characteristic necrotizing features. An accurate distinction can be made on the basis of a multidisciplinary approach based on a clinical evaluation, laboratory workout, analysis of radiological findings and finally bioptic sampling. It is also important to stain pathology for micro-organisms to exclude the most frequent infections that could cause similar histopathological and clinical pictures [77]. Some examples of sarcoidosis-like conditions can be the following:

### 4.1. Hypersensitivity Pneumonitis (HP) or Extrinsic Allergic Alveolitis

HP is a chronic granulomatous pulmonary disease triggered by the inhalation of organic substances that act as antigens such as bacteria, fungi, drugs or animal-derived proteins. The characteristic features are the presence of non-caseous epithelioid granulomas smaller than those of sarcoidosis at biopsy. Unlike sarcoidosis, the hilar lymph nodes are not affected, and granulomas are mainly located in the peribronchiolar region. The alveolar septa present infiltrates consisting of macrophages, lymphocytes and plasma cells. There is bronchiolar obstruction correlated to the ulceration of the surface coating. Foamy macrophages are present in the alveolar lumen and, in some cases, Schaumann bodies are present in the giant cells of the granuloma. The bronchoalveolar lavage can give some diagnostic clues because CD8+ suppressor lymphocytes are predominant in hypersensitivity pneumonitis [77].

### 4.2. Berylliosis

The granulomatous reaction to Beryllium is caused by the activation of CD4+ lymphocytes that recognize Beryllium and produce IL-2 and IFN gamma, activating the multinucleated phagocytes and starting the formation of granulomas in the interstitium and in the pulmonary lymph nodes. The granuloma in berylliosis is indistinguishable from those found in sarcoidosis, the main structure is always a non-caseous granuloma consisting of epithelioid and Langherans cells with areas of hyalinosis and bodies including asteroids. A history of beryllium exposition or the demonstration of the presence of beryllium in biological fluids or tissues are diagnostics of berylliosis [78].

### 4.3. Granulomatosis with Polyangiitis (GPA, Formerly Wegner’s Granulomatosis)

GPA is a systemic disease characterized by necrotizing granulomatous lesions affecting both the upper and lower airways. Unlike sarcoidosis, GPA has no distinct granulomas, multinucleated giant cells are present in low quantity and the area of necrosis has irregular contours similar to a geographical map, with a large amount of cellular debris and it is surrounded by histiocytes. Other typical features are the presence of a necrotizing vasculitis with fibrinoid necrosis of the media of the affected vessels and that granulomas, unlike other granulomatous diseases, do not affect the lymph nodes [79].

### 4.4. Fungal Infections

Various fungal infections such as Aspergillosis, Coccidioidomycosis, Blastomycosis, Histoplasmosis can produce granulomatous reactions in the lungs that enter into differential diagnosis with sarcoidosis. Unlike sarcoidosis, the granulomas of these fungal infections most often present suppurative necrosis [80].

Occasionally, Histoplasma may not be visible in granulomas through the usual hematoxylin-eosin staining, unlike other fungal infections, and often it is necessary to use the Silver methenamine, Giemsa or periodic acid-Schiff (PAS) staining [81].

### 4.5. Granulomas from Mycobacterial Tuberculosis

The histopathological features of M. tuberculosis, both in the miliary and in the isolated nodular form, often pose significant issues in the differential diagnosis of sarcoidosis. The histological differentiation between sarcoid granuloma and tuberculoma is based on the characteristics of the tuberculous granuloma [82]. In its typical form it has a more relevant lymphocytes wall and a larger amount of multinucleated giant cells (located mainly in the central area). Furthermore, the mycobacteria present in the ‘core’ of the granuloma are alcohol and acid resistant, and there is little peripheral delimitation of the granuloma with a tendency for two or more tuberculomas to merge together; in the majority of cases, tuberculomas have a coagulative necrotizing evolution toward a caseous nature [81].

In a large case series, a Ziehl–Neelsen stain demonstrated the presence of acid-fast organisms in approximately 77% of solitary necrotizing granulomas, but showed a low accuracy to identify the presence of these organisms in non-necrotizing or hyalinized granulomas. In these cases, sarcoid granulomas can be differentiated with difficulty [83].

The history of potential exposure to bacilli, diagnostic imaging showing suggestive patterns of tuberculosis and also the gene amplification tests can orient toward the correct diagnosis. The Xpert MTB/RIF assay is a novel semi-automatic DNA amplification assay that can simultaneously identify M. tuberculosis and rifampicin resistance [84].

### 4.6. Non-Tuberculous Mycobacterial (NTM) Granulomas

Other forms of non-tuberculous mycobacterial infection can produce granulomas, especially in the lung or skin, similar to those of sarcoidosis. Mycobacteria mainly involving the lungs are M. avium, M. complex and M. Kansasii. Traditionally it was thought that NTM infection of the lungs was associated with immunodeficiency or pre-existing lung disease. However, it is now recognized that NTM infection of the lungs also occurs in immunocompetent and healthy patients [85].

In immunocompromised patients, NTM infection is characterized by ill-defined granulomas consisting of foamy macrophages containing mycobacteria [86]. In immunocompetent patients, NTM infection can cause the formation of both necrotizing and non-necrotizing granulomas in the lungs in the peribronchiolar region [87].

### 4.7. Foreign Body Granuloma

Histologically, foreign body granulomas typically consist of multinucleated giant cells and macrophages. These giant cells are typically of the “foreign body type” and have nuclei scattered irregularly throughout the cytoplasm [88].

**Figure 6:**
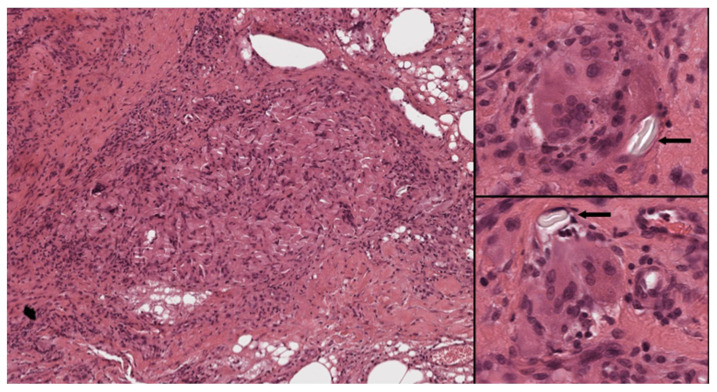
Foreign body reaction to suture material. Left: Histiocytes are deeply intermingled with the suture material. Right: Numerous foreign body-type giant cells are seen around inert material (arrows) (Left: H&E 40×; right, top and bottom: H&E 400×).

In many cases the foreign body can be recognized within these granulomas (Figure 6). These granulomas can develop in the skin due to the penetration of foreign bodies following trauma or injection of substances, for example in tattoos. Foreign body granulomas can form near the airways in the case of aspiration of heterologous material, in the perivascular site in the case of intravenous injection of foreign bodies (e.g. drugs) or in the interalveolar areas following inhalation of fibrous substances [89].

### 4.8. Sarcoid-Like Reaction from Drug and Malignancies

Several drugs have been associated with the onset of granulomatous inflammation that is histologically indistinguishable from the reaction observed in patients with sarcoidosis. Interferons, tumor necrosis factor-α, the combination of antiretroviral therapies, monoclonal antibodies, immune checkpoint inhibitors and other immunosuppressive agents are just a few drugs which can be the cause of granulomatous formation, effect of a drug-induced alteration of the immune response [90,91]. Although granulomas resolve after drug discontinuation, the clinical picture is usually mild, thus interruption of drug treatment is not always necessary. It is currently unknown if the sarcoid-like reaction is a distinctive disorder or a real sarcoidosis-induced condition from drugs [90]. Also, some malignant tumors can trigger a significant granulomatous reaction [31], above all 13.8% of Hodgkin’s lymphomas, 7.3% of non-Hodgkin disease and 4.4% of all carcinomas (e.g., lung adenocarcinoma), being otherwise rare in other malignant tumors such as sarcomas. It is hypothesized that tumor cell antigens could be the main triggers for the immune reaction associated with the sarcoid-like granulomas, and the distinction of the two disorders is often difficult due to the overlap of clinical and imaging features, except for the histopathological findings [92]. Instead, the differentiation between sarcoid-like reaction induced by malignancies and drugs from real sarcoidosis is often difficult or downright impossible. However, unlike sarcoidosis, B-cell positive granulomas have been found in malignant tumors and drug-induced sarcoid like reactions, and this feature could be useful to differentiate between the two conditions [93].

## 5. Conclusions

In most of the cases, the diagnosis of sarcoidosis is achieved by obtaining a sample biopsy of the affected tissue and by recognizing the presence of the characteristic non-caveating and non-necrotizing granulomas. However, histopathology is not always pathognomonic since these findings can be found also in other granulomatous disorders. A thorough assessment of the case, by considering all the aspects of the disease including clinical, radiological and histopathological findings, is mandatory to achieve a correct diagnosis, and to differentiate sarcoidosis from the other granulomatous-related diseases.

## Figures and Tables

**Table 1:** Scadding classification of lung involvement from sarcoidosis at chest X-ray.

Stage	Chest X-Ray Findings	Frequency (%)	Resolution (%)
0	Normal	5–15	
I	Bilateral hilar lymph node enlargement	25–65	60–90
II	Bilateral hilar lymph node enlargement and lung infiltration	20–40	40–70
III	Lung infiltration without lymph node enlargement	10–15	10–20
IV	Lung fibrosis	5	0

## Data Availability

Not applicable.

## References

[1] Tana C. (2021). Sarcoidosis: An Old but Always Challenging Disease. Diagnostics.

[2] Arkema E.V., Cozier Y.C. (2020). Sarcoidosis epidemiology: Recent estimates of incidence, prevalence and risk factors. Curr. Opin. Pulm. Med..

[3] Tana C., Mantini C., Donatiello I., Mucci L., Tana M., Ricci F., Cipollone F., Giamberardino M.A. (2021). Clinical Features and Diagnosis of Cardiac Sarcoidosis. J. Clin. Med..

[4] Hena K.M. (2020). Sarcoidosis Epidemiology: Race Matters. Front. Immunol..

[5] Prasse A., Katic C., Germann M., Buchwald A., Zissel G., Müller-Quernheim J. (2008). Phenotyping sarcoidosis from a pulmonary perspective. Am. J. Respir. Crit. Care Med..

[6] Denny M.C., Fotino A.M. (2013). The Heerfordt-Waldenström syndrome as an initial presentation of sarcoidosis. Bayl. Univ. Med. Cent. Proc..

[7] Chokoeva A.A., Tchernev G., Tana M., Tana C. (2014). Exclusion criteria for sarcoidosis: A novel approach for an ancient disease?. Eur. J. Intern. Med..

[8] Chappell A.G., Cheung W.Y., Hutchings H.A. (2000). Sarcoidosis: A long-term follow up study. Sarcoidosis Vasc. Diffus. Lung Dis..

[9] Neville E., Walker A.N., James D.G. (1983). Prognostic factors predicting the outcome of sarcoidosis: An analysis of 818 patients. Q. J. Med..

[10] Judson M.A., Baughman R.P., Teirstein A.S., Terrin M.L., Yeager H. (1999). Defining organ involvement in sarcoidosis: The ACCESS proposed instrument. ACCESS Research Group. A Case Control Etiologic Study of Sarcoidosis. Sarcoidosis Vasc. Diffuse. Lung Dis..

[11] Judson M.A., Costabel U., Drent M., Wells A., Maier L., Koth L., Shigemitsu H., Culver D.A., Gelfand J., Valeyre D. (2014). Organ Assessment Instrument Investigators TW. The WASOG Sarcoidosis Organ Assessment Instrument: An update of a previous clinical tool. Sarcoidosis Vasc. Diffus. Lung Dis..

[12] Katsushika S., Kodera S., Nakamoto M., Ninomiya K., Kakuda N., Shinohara H., Matsuoka R., Ieki H., Uehara M., Higashikuni Y. Deep Learning Algorithm to Detect Cardiac Sarcoidosis From Echocardiographic Movies. Circ. J..

[13] Tana C., Mantini C., Cipollone F., Giamberardino M.A. (2021). Chest Imaging of Patients with Sarcoidosis and SARS-CoV-2 Infection. Current Evidence and Clinical Perspectives. Diagnostics (Basel).

[14] Lynch J.P., Kazerooni E.A., Gay S.E. (1997). Pulmonary sarcoidosis. Clin. Chest Med..

[15] McKinzie B.P., Bullington W.M., Mazur J.E., Judson M.A. (2010). Efficacy of short-course, low-dose corticosteroid therapy for acute pulmonary sarcoidosis exacerbations. Am. J. Med. Sci..

[16] Rajesh S., Randeep G., Anant M., Chinmoyee D. (2004). Scadding Criteria for Diagnosis of Sarcoidosis: Is There A Need For Change?. Chest.

[17] Raoof S., Amchentsev A., Vlahos I., Goud A., Naidichm D.P. (2006). Pictorial essay: Multinodular disease: A high-resolution CT scan diagnostic algorithm. Chest.

[18] Nishino M., Lee K.S., Itoh H., Hatabu H. (2010). The spectrum of pulmonary sarcoidosis: Variations of high-resolution CT findings and clues for specific diagnosis. Eur. J. Radiol..

[19] Polychronopoulos V.S., Prakash U.B. (2009). Airway involvement in sarcoidosis. Chest.

[20] Rosen Y., Dail D.H., Hammar S.P. (1994). Sarcoidosis. Pulmonary Pathology.

[21] Nardi A., Brillet P.-Y., Letoumelin P., Girard F., Brauner M., Uzunhan Y., Naccache J.-M., Valeyre D., Nunes H. (2011). Stage IV sarcoidosis: Comparison of survival with the general population and causes of death. Eur. Respir. J..

[22] Smith L.J., Lawrence J.B., Katzenstein A.A. (1983). Vascular sarcoidosis: A rare cause of pulmonary hypertension. Am. J. Med. Sci..

[23] Rafferty P., Biggs B.A., Crompton G.K., Grant I.W. (1983). What happens to patients with pulmonary aspergilloma? Analysis of 23 cases. Thorax.

[24] Dana M.R., Merayo-Lloves J., Schaumberg D.A., Foster C.S. (1996). Prognosticators for visual outcome in sarcoid uveitis. Ophthalmology.

[25] Ohara K., Judson M.A., Baughman R.P. (2005). Clinical aspects of ocular sarcoidosis. Eur. Respir. J. Monog..

[26] Hart L.A., Conron M., du Bois R.M. (2001). Sarcoidosis. Int. J. Tuberc. Lung Dis..

[27] Henry K., Symmers W.S.C. (1993). Thymus, Lymph Nodes, Spleen and Lymphatics.

[28] Goble R.R., Murray P.I. (1995). Fuch’s heterochromic uveitis and sarcoidosis. Br. J. Ophthalmol..

[29] Yanardag H., Pamuk O.N. (2003). Lacrimal gland involvement in sarcoidosis. The clinical features of 9 patients. Swiss Med. Wkly..

[30] Tchernev G., Cardoso J.C., Chokoeva A.A., Verma S.B., Tana C., Ananiev J., Gulubova M., Philipov S., Kanazawa N., Nenoff P. (2014). The “mystery” of cutaneous sarcoidosis: Facts and controversies. Int. J. Immunopathol. Pharmacol..

[31] Tchernev G., Tana C., Schiavone C., Cardoso J.C., Ananiev J., Wollina U. (2014). Sarcoidosis vs. Sarcoid-like reactions: The Two Sides of the same Coin?. Wien Med. Wochenschr..

[32] Eklund A., Rizzato G. (2005). Skin manifestations in sarcoidosis. Eur. Resp. J. Monog..

[33] Requena L., Requena C. (2003). Erythema Nodosum. Dermat. Online J..

[34] Chao S.C., Yan J.J., Lee J.Y. (2000). Cutaneous sarcoidosis among Taiwanese. J. Formos. Med. Assoc..

[35] Iannuzzi M.C., Rybicki B.A., Teirstein A.S. (2007). Sarcoidosis. N. Engl. J. Med..

[36] Dubrey S.W., Bell A., Mittal T.K. (2007). Sarcoid heart disease. Postgrad. Med. J..

[37] Sharma O.P. (2003). Diagnosis of cardiac sarcoidosis: An imperfect science, a hesitant art. Chest.

[38] Yazaki Y., Isobe M., Hiroe M., Morimoto S., Hiramitsu S., Nakano T., Izumi T., Sekiguchi M., Central Japan Heart Study Group (2001). Prognostic determinants of long-term survival in Japanese patients with cardiac sarcoidosis treated with prednisone. Am. J. Cardiol..

[39] Yigla M., Badarna-Abu-Ria N., Tov N., Ravell-Weiller D., Rubin A.-H.E. (2002). Sarcoidosis in northern Israel; clinical character-istics of 120 patients. Sarcoidosis Vasc. Diffus. Lung Dis. Off. J. WASOG World Assoc. Sarcoidosis Granulomatous Disord..

[40] Baughman R.P., Teirstein A.S., Judson M.A., Rossman M.D., Yeager H., Bresnitz E.A., DePalo L., Hunninghake G., Iannuzzi M.C., Johns C.J. (2001). Clinical characteristics of patients in a case control study of sarcoidosis. Am. J. Respir. Crit. Care Med..

[41] Harder H., Büchler M.W., Fröhlich B., Ströbel P., Bergmann F., Neff W., Singer M.V. (2007). Extrapulmonary sarcoidosis of liver and pancreas: A case report and review of literature. World J. Gastroenterol..

[42] Tana C., Silingardi M., Dietrich C.F. (2015). New trends in ultrasound of hepatosplenic sarcoidosis. Z Gastroenterol..

[43] Lehmuskallio E., Hannuksela M., Halme H. (1977). The liver in sarcoidosis. Acta Med. Scand..

[44] Thudani U., Aber C.P., Taylor J.J. (1975). Massive splenomegaly, pancytopenia and hæmolitic anaemia in sarcoidosis. Acta Haematol..

[45] Tana C., Donatiello I., Coppola M.G., Ricci F., Maccarone M.T., Ciarambino T., Cipollone F., Giamberardino M.A. (2020). CT Findings in Pulmonary and Abdominal Sarcoidosis. Implications for Diagnosis and Classification. J. Clin. Med..

[46] Tana C., Iannetti G., Mezzetti A., Schiavone C. (2014). Splenic sarcoidosis remains a diagnostic challenge. J. Clin. Ultrasound..

[47] Tana C., Iannetti G., D’Alessandro P., Tana M., Mezzetti A., Schiavone C. (2013). Pitfalls of contrast-enhanced ultrasound (CEUS) in the diagnosis of splenic sarcoidosis. J. Ultrasound..

[48] Tana C., Dietrich C.F., Schiavone C. (2014). Hepatosplenic sarcoidosis: Contrast-enhanced ultrasound findings and implications for clinical practice. Biomed Res. Int..

[49] Tana C., Wegener S., Borys E., Pambuccian S., Tchernev G., Tana M., Giamberardino M.A., Silingardi M. (2015). Challenges in the diagnosis and treatment of neurosarcoidosis. Ann. Med..

[50] Nozaki K., Scott T.F., Sohn M., Judson M.A. (2012). Isolated neurosarcoidosis: Case series in 2 sarcoidosis centers. Neurologist.

[51] Hoitsma E., Faber C.G., Drent M., Sharma O.P. (2004). Neurosarcoidosis: A clinical dilemma. Lancet Neurol.

[52] Stern B.J., Krumholz A., Johns C., Scott P., Nissim J. (1985). Sarcoidosis and its neurological manifestations. Arch. Neurol..

[53] Nemni R., Galassi G., Cohen M., Hays A.P., Gould R., Singh N., Bressman S., Gamboa E.T. (1981). Symmetric sarcoid polyneu-ropathy: Analysis of a sural nerve biopsy. Neurology.

[54] Murialdo G., Tamagno G. (2002). Endocrine aspects of neurosarcoidosis. J. Endocrinol. Investig..

[55] Barnard J., Newman L.S. (2001). Sarcoidosis: Immunology, rheumatic involvement, and therapeutics. Curr. Opin. Rheumatol..

[56] Flipo R.M., Cotton A. (1995). Sarcoidosic dactylitis. Rev. Med. Intern..

[57] Neville E., Carstairs L.S., James D.G. (1977). Sarcoidosis of bone. Q. J. Med..

[58] Mañá J., Gómez-Vaquero C., Montero A., Salazar A., Marcoval J., Valverde J., Manresa F., Pujol R. (1999). Löfgren’s syndrome revisited: A study of 186 patients. Am. J. Med..

[59] Awada H., Abi-Karam G., Fayad F. (2003). Musculoskeletal and other extrapulmonary disorders in sarcoidosis. Best Pract. Res. Clin. Rheumatol..

[60] Bhargava V., Farhi D.C. (1988). Bone marrow granulomas: Clinicopathologic findings in 72 cases and review of the literature. Hematol. Pathol..

[61] Brackers de Hugo L., French M., Broussolle C., Sève P. (2013). Granulomatous lesions in bone marrow: Clinicopathologic findings and significance in a study of 48 cases. Eur. J. Intern. Med..

[62] Peña-Garcia J.I., Shaikh S., Barakoti B., Papageorgiou C., Lacasse A. (2019). Bone marrow involvement in sarcoidosis: An elusive extrapulmonary manifestation. J. Community Hosp. Intern. Med. Perspect..

[63] Berliner A.R., Haas M., Choi M.J. (2006). Sarcoidosis: The nephrologist’s perspective. Am. J. Kidney Dis..

[64] Göbel U., Kettritz R., Schneider W., Luft F.C. (2001). The Protean face of renal sarcoidosis. J. Am. Soc. Nephrol..

[65] Costabel U., Ohsimo S., Guzman J. (2008). Diagnosis of sarcoidosis. Curr. Opin. Pulm. Med..

[66] Semenzato G., Pezzutto A., Chilosi M., Pizzolo G. (1982). Redistribution of T-lymphocytes in the lymph nodes of patients with sarcoidosis. N. Engl. J. Med..

[67] Gal A.A., Koss M.N. (2002). The pathology of sarcoidosis. Curr. Opin. Pulm. Med..

[68] Tana C., Giamberardino M.A., Di Gioacchino M., Mezzetti A., Schiavone C. (2013). Immunopathogenesis of sarcoidosis and risk of malignancy: A lost truth?. Int. J. Immunopathol. Pharmacol..

[69] Reid J.D., Andersen M.E. (1988). Calcium oxalate in sarcoid granulomas. Am. J. Clin. Pathol..

[70] Rosen Y. (2007). Pathology of sarcoidosis. Semin Respir. Crit. Care Med..

[71] Jones Williams W. (1960). The nature and origin of Schaumann bodies. J. Pathol. Bacteriol..

[72] Boutet M. (1975). Ultrastructural and histochemical study of Hamasaki-Wesenberg bodies in lymphonode sarcoidosis. Ann. Anat. Pathol..

[73] Liebow A.A. (1973). The J. Burns Amberson lecture–pulmonary angiitis and granulomatosis. Am. Rev. Respir. Dis..

[74] Karpathiou G., Batistatou A., Boglou P., Stefanou D., Froudarakis M.E. (2018). Necrotizing sarcoid granulomatosis: A distinctive form of pulmonary granulomatous disease. Clin. Respir. J..

[75] Le Gall F., Loeuillet L., Delaval P., Thoreux P.H., Desrues B., Ramee M.P. (1996). Necrotizing sarcoid granulomatosis with and without extrapulmonary involvement. Path. Res. Pract..

[76] Tana C., Schiavone C. (2021). The Chameleon Behavior of Sarcoidosis. J. Clin. Med..

[77] Coleman A., Colby T.V. (1988). Histologic diagnosis of extrinsic allergic alveolitis. Am. J. Surg. Pathol..

[78] Popper H.H. (1999). Epithelioid cell granulomatosis of the lung: New insights and concepts. Sarcoidosis Vasc. Diffus. Lung Dis..

[79] Godman G., Churg J. (1954). Wegener’s granulomatosis. A.M.A. Arch. Pathol..

[80] Mukhopadhyay S. (2001). Role of histology in the diagnosis of infectious causes of granulomatous lung disease. Curr. Opin. Pulm. Med..

[81] Mukhopadhyay S., Gal A.A. (2010). Granulomatous lung disease: An approach to the differential diagnosis. Arch. Pathol. Lab. Med..

[82] Arar O., Boni F., Meschi T., Tana C. (2019). Pulmonary Sarcoidosis Presenting with Miliary Opacities. Curr. Med. Imaging Rev..

[83] Hsu R.M., Connors A.F., Tomashefski J.F. (1996). Histologic, microbiologic, and clinical correlates of the diagnosis of sarcoi-dosis by transbronchial biopsy. Arch. Pathol. Lab. Med..

[84] Boehme C.C., Nabeta P., Hillemann D., Nicol M., Shenai S., Krapp F., Allen J., Tahirli R., Blakemore R., Rustomjee R. (2010). Rapid molecular detection of tuberculosis and rifampin resistance. N. Engl. J. Med..

[85] Griffith D.E., Aksamit T., Brown-Elliott B.A., Catanzaro A., Daley C., Gordin F., Holland S.M., Horsburgh R., Huitt G., Iademarco M.F. (2007). An official ATS/IDSA statement: Diagnosis, treatment, and prevention of nontuberculous mycobacterial diseases. Am. J. Respir. Crit. Care Med..

[86] Wallace J.M., Hannah J.B. (1988). Mycobacterium avium complex infection in patients with the acquired immunodeficiency syn-drome. A clinicopathologic study. Chest.

[87] Corpe R.F., Runyon E.H., Lester W. (1963). Status of disease due to unclassified mycobacteria. A statement of the Subcommittee on Unclassified Mycobacteria of the Committee on Therapy. Am. Rev. Respir. Dis..

[88] Weeden D. (2010). The Granulomatous Reaction Pattern. Weedon’s Skin Pathology.

[89] Churg C., Green H.Y., Churg A., Green F.Y. (1988). Miscellaneous conditions. Pathology of Occupational Lung Disease.

[90] Miedema J., Nunes H. (2021). Drug-induced sarcoidosis-like reactions. Curr. Opin. Pulm. Med..

[91] Cabanié C., Ammari S., Hans S., Pobel C., Laparra A., Danlos F.X., Chanson N., Dolidon S., Seban R., Voisin A.L. (2021). Outcomes of patients with cancer and sarcoid-like granulomatosis associated with immune checkpoint inhibitors: A case-control study. Eur. J. Cancer..

[92] Brincker H. (1986). Sarcoid reactions in malignant tumours. Cancer Treat Rev..

[93] Brincker H., Pedersen N.T. (1991). Immunohistologic separation of B-cell-positive granulomas from B-cell-negative granulomas in paraffin-embedded tissues with special reference to tumor-related sarcoid reactions. APMIS.

